# Effects of Chemical Composition and Cross-Linking Degree on the Thermo-Mechanical Properties of Bio-Based Thermosetting Resins: A Molecular Dynamics Simulation Study

**DOI:** 10.3390/polym16091229

**Published:** 2024-04-28

**Authors:** Qiuyu Tang, Jie Jiang, Jinjin Li, Ling Zhao, Zhenhao Xi

**Affiliations:** 1 State Key Laboratory of Chemical Engineering, School of Chemical Engineering, East China University of Science and Technology, Shanghai 200237, China; qiuyu_hl@163.com (Q.T.); lijinjin@ecust.edu.cn (J.L.); zhaoling@ecust.edu.cn (L.Z.); 2 Shanghai Key Laboratory of Advanced Polymeric Materials, School of Materials Science and Engineering, East China University of Science and Technology, Shanghai 200237, China; 3 Shanghai Key Laboratory of Multiphase Materials Chemical Engineering, East China University of Science and Technology, Shanghai 200237, China

**Keywords:** bio-based thermoset, MD simulation, cross-linking

## Abstract

Bio-based epoxy resins have received significant attention in terms of concerns regarding carbon emission. Epoxidized soybean oil (ESO) derived from sustainable feedstock has been widely used to blend with traditional diglycidyl ether of bisphenol-A (DGEBA) to replace some of the petroleum-based components. In this work, molecular dynamics (MD) simulations were applied to track the network formation and predict the performance of methyl hexahydrophthalic anhydride (MHHPA)-cured ESO/DGEBA blend systems. The effects of ESO content and cross-linking degree on the mass density, volumetric shrinkage, glass transition temperature (*T*_g_), coefficient of thermal expansion (CTE), Young’s modulus, yield strength, and Poisson’s ratio of the epoxy resin were systematically investigated. The results show that systems with high ESO content achieve gelation at low cross-linking degree. The *T*_g_ value, Young’s modulus, and yield strength increase with the increase in cross-linking degree, but the CTE at the glassy state and Poisson’s ratio decrease. The comparison results between the simulated and experimental data demonstrated that the MD simulations can accurately predict the thermal and mechanical properties of ESO-based thermosets. This study gains insight into the variation in thermo-mechanical properties of anhydride-cured ESO/DGEBA-based epoxy resins during the cross-linking process and provides a rational strategy for optimizing bio-based epoxy resins.

## 1. Introduction

Chemical industry is encouraged to transition towards sustainable chemistry due to environmental pollution, depletion of petroleum resources, and urgency for low-carbon economic development. Efforts to develop more environmentally friendly alternatives to traditional petroleum-based epoxy resins (ERs) have led to the increasing popularity of bio-based ERs [1]. Soybean oil (SO) is extensively used as a substitute for the petroleum-based ERs due to its low price, wide availability, and non-toxicity, along with its ability to be modified by epoxidation or acrylation [2,3]. Epoxidized soybean oils (ESO) are generally used as either plasticizers or stabilizers for polyvinyl chloride (PVC) materials, paints, or coatings [4]. They can also be reacted with amines [5], anhydrides [6], and acids [7] to prepare thermosetting polymers. Several researchers [8,9,10] have reported the successful synthesis and characterization of ESO-based thermosets. Due to the flexibility of the long carbon chains in the ESO structure, ESO-based thermosets are rarely used alone in engineering applications. Instead, they are prepared as composites with carbon nanotubes [11], cellulose [12], etc. Additionally, ESO can be blended with diglycidyl ether of bisphenol-A (DGEBA) to synthesize thermosetting resins because of its good miscibility with DGEBA. Altuna [13,14] and Kumar [15,16] et al. have reported the synthesis of ERs using ESO to replace the DGEBA resin with methylhexahydrophthalic anhydride (MHHPA) as a cross-linking agent. The results proved that the addition of ESO not only enhanced the bio-based content of the resin but also improved its tensile strength, impact strength, and flexibility. ESO/DGEBA hybrid epoxy resins are recognized to have great potential for industrial applications. Therefore, it is essential to understand the relationships between the structure and properties of the resin to produce and optimize its properties.

Recently, a molecular dynamics (MD) simulation technique has received much attention in the field of polymer design. MD provides insightful information at the atomic and micro length scales that are usually not obtained from experiments. This approach enables the prediction of the thermo-mechanical properties of epoxy resins, a thorough comprehension of network structure and the mechanisms of thermo-mechanical property formation [17,18,19,20,21]. Varshney et al. [22] proposed a multistep relaxation procedure for relaxing the molecular topology during cross-linking and predicted the material properties of epoxy-based networks (EPON-862/DETDA) such as density, glass transition temperature, and thermal expansion coefficient, which were found to be in good agreement with experimental results. Odegard et al. [23,24] implemented reactive interface force field (IFF-R) to accurately predict the physical and mechanical properties of an epoxy system at varying degrees of cure ranging from fully uncross-linked to fully cross-linked states at room temperature. Radue et al. [25] has revealed that monomer functionality has a significant influence on the Young’s modulus and yield stress of cross-linked materials using Reax Force Field (ReaxFF). The Young’s modulus of tri-functional and tetra-functional epoxy resins was found to be higher than that of bi-functional epoxy resins. Wang et al. [26] used MD to elucidate the chemical composition and cross-link effects on the linear and nonlinear viscoelasticity of the ethylene propylene diene monomer (EPDM) elastomer under different strain rates. Shundo et al. [27] studied the effect of curing temperature on fracture behavior of DGEBA and n-alkyl diamine epoxy resins.

Recently, the prediction of thermo-mechanical properties of anhydride-cured epoxy resin systems has also aroused increasing interest [28,29]. Fu et al. [30] revealed that the slight structure difference between ethyl tetrahydrophthalic anhydride (MTHPA) and nadic anhydride (NA) has a significant effect on the synergy rotational energy barrier, cohesive energy density, and fraction free volume, thus affecting the *T*_g_ and the modulus. However, there are few reports on the performance predictions and structural property relationships for anhydride-cured epoxidized vegetable oil-based thermosets. The reported experimental studies mainly focused on characterizing completely cured epoxy resins. There are still challenges in identifying the evolution of their structure and properties during the curing process. Thus, it is important to elucidate the complex relationship between the molecular structure, including the network, and the characteristic components and mechanical properties. 

In this work, a molecular simulation study aiming at predicting the properties of a bio-based and petroleum-blend thermosetting system was presented. A modeling approach was applied to study the epoxy system containing different ratios of ESO and DGEBA cured with MHHPA. The radial distribution function (RDF), density, fraction of free volume, thermal response, and mechanical response during the cross-linking process were obtained by MD simulation. The influences of the ESO content and the cross-linking degree on the thermosetting properties were discussed.

## 2. Methodology

### 2.1. Monomer Structures

**Figure 1:**
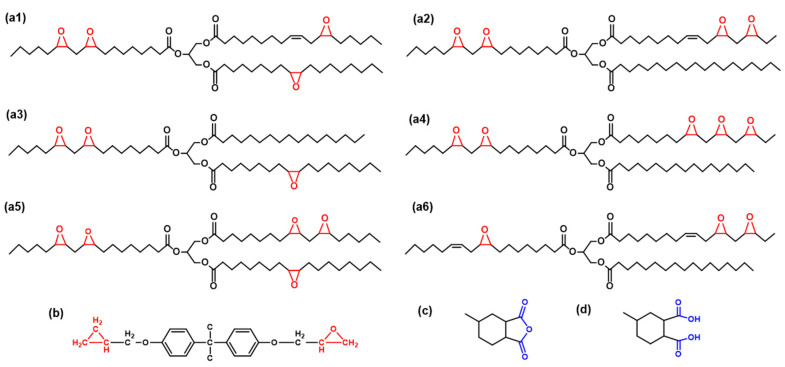
The molecular structure of (**a1**–**a6**) ESO, (**b**) DGEBA, (**c**) MHHPA, and (**d**) MHHPA-OH.

The molecular structure of soybean oil comprises [31] of linoleic acid (51%), oleic acid (25%), palmitic acid (11%), linolenic acid (9%), and stearic acid (4%). To replicate as accurately as possible the actual composition of epoxidized soybean oil, we created six molecular structures as depicted in Figure 1(a1–a6). The ESO used in the simulation is a blend of these six structures, with an average epoxy functionality of 4, consistent with commercial ESO. Figure 1b and Figure 1c show the molecular structures of DGEBA, MHHPA, and MHHPA-OH, respectively.

### 2.2. Simulation Details

The modeling of ESO, DGEBA, MHHPA, MHHPA-OH, and the cross-link network was established by Materials studio 2016 software (Accelrys Inc., San Diego, CA, USA). The composition of the simulation system is shown in Table 1. Molecular dynamics simulations were performed using a large-scale/molecular massively parallel simulator [32] (LAMMPS 3 March 2020) MD software package with the second-generation COMPASS II force field [33] (condensed-phase optimized molecular potentials for atomistic simulation studies), which has been shown to provide accurate predictions of thermo-mechanical properties of thermosetting polymers. The Nosé–Hoover [34] thermostat and barostat were used to control temperature and pressure, respectively. The fluctuation times of the thermostat and barostat (T_damp_ and P_damp_) were 50 and 500 time steps, respectively. The cut-off distance for electrostatic and van der Waals forces was set as 10 Å, while the electrostatic force was calculated using the Ewald method.

### 2.3. Modeling

**Figure 2:**
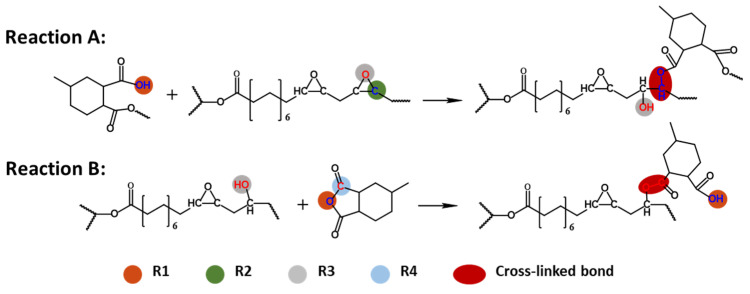
Curing paths of anhydride-cured epoxy and setting of reactive atom pairs.

The modeling of cross-linking structure is based on the reaction path, involving two main reactions, as shown in Figure 2. The first reaction is a ring-opening reaction between the carboxyl and epoxy groups, resulting in the formation of ester and hydroxyl groups (Reaction A). The initial carboxyl group in the system comes from the addition of MHHPA-OH. The hydroxyl group from Reaction A then reacts with the anhydride group to form an ester group and a new reactive carboxyl group (Reaction B). These two reactions alternate to form a cross-linked network. The reaction sites of the monomer and cross-linker are labeled according to the curing path. The “O” atoms of the anhydride group (red balls) and the “C” atoms of the epoxy group in monomer (green balls) are labeled as R1 and R2, respectively. The “O” atoms of the epoxy group in the monomer (gray balls) and the “C” atoms of the anhydride group in the cross-linker (blue balls) are labeled as R3 and R4, respectively. Additionally, R1 is exclusively activated on the carboxyl group of the anhydride following the ring-opening process, while R3 is activated on the hydroxyl group after the epoxy ring-opening. During the modeling process, two pairs of reaction atoms, R1/R2 and R3/R4, were detected.

This approach to constructing cross-linked DGEBA/ESO-based networks relies on spatial proximity as a criterion for bond formation. Reaction sites were designated on the R1–R2 and R3–R4. Notably, R1 and R3 atoms were only considered activated atoms after the hydroxyl group has formed. Reaction occurs between a pair of reactive atoms (R1/R2 or R3/R4) when their distance was within the reaction cut-off distance. The range of cut-off distance was determined as 4–9 Å, and the cut-off step was set as 0.5 Å. The specific cross-linking simulation protocol is described in detail in the following simulation flow.

1. The optimized ESO, DGEBA, MHHPA, and MHHPA-OH molecules were randomly packed into a periodic boundary cubic box according to the composition listed in Table 1. And the composition of the ESO is shown in Table S1 and Figure S1 in the Supporting Information. The initial density was set to 0.2 g/cm^3^ and then gradually compressed a target mass density under cross-linking conditions (500 K, 10 atm). After reaching the target density, the models were annealed in the NVT ensemble by ramping up to an elevated temperature (300–600 K). The annealing process was followed by an equilibration sequentially in the NVT ensemble and in the NPT ensemble for 200 ps.
2. After equilibration, the root-mean-squared (RMS) distances between actived-R1 and R2 and that of between actived-R3 and R4 were computed. The minimum RMS distance pair was then selected, and a bond was formed if this distance was less than the upper-bound cut-off. Then, the atomic information associated with the reacting atoms and the topology of the system were immediately updated. Energy minimization was performed after each successful cross-link. After each cycle, the structure was equilibrated in the NPT system for 50 ps. Both energy minimization and equilibration were performed to remove residual stress generated due to cross-linking. The procedure continued until the desired conversion rate was reached.
3. Following the cross-linking, models with cross-linking degrees of 50%, 60%, 70%, 80%, and 85% were chosen. An NVT simulation and an NPT simulation were performed for each cross-linking degree at 500 K for 2 ns to relax the model and to predict the final density and volume. Here, the cross-linking degree was calculated according to Equation (1).

(1) $$\mathrm{Cross}\text{-}\mathrm{linking}\;\mathrm{degree}=\frac{\mathrm{Number}\;\mathrm{of}\;\mathrm{cross}\text{-}\mathrm{links}}{2\;\times \;\mathrm{Total}\;\mathrm{numbers}\;\mathrm{of}\;\mathrm{epoxy}\;\mathrm{group}}$$

For each sample, three independent replicates were generated to obtain the mean and the standard deviation of the performance prediction.

### 2.4. Cooling Process

**Figure 3:**
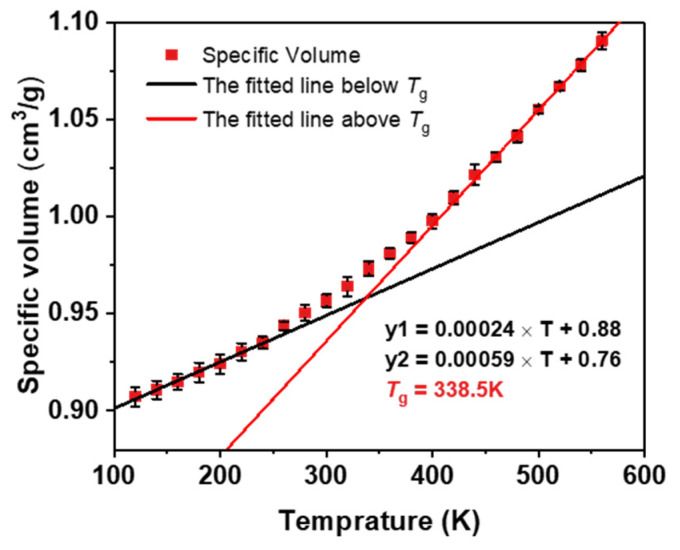
Specific volume–temperature relationships for the ESO100-85% system.

In order to obtain the glass transition temperature (*T*_g_) of the system, stepwise cooling simulations from 580 K to 120 K were performed on the cross-linked system. Dynamic simulations were conducted for 600 ps at each temperature point under the NPT ensemble, resulting in a cooling rate of 20 K/600 ps. The specific volume values for each temperature are the average of three replicates that were cooled down twice. Figure 3 shows the specific volume–temperature relationship of ESO100 at 85% cross-linking degree. It can be clearly seen that the specific volume of ESO100-85% in the rubbery and glassy states has a bilinear response with different slopes with respect to temperature. The *T*_g_ value is defined as the temperature corresponding to the intersection of the two slopes. The coefficient of volumetric thermal expansion (CTE) was calculated using Equation (2):

(2) $$\alpha =\frac{1}{V_{0}}{\left(\frac{\partial V}{\partial T}\right)}_{P}$$

*α* is the coefficient of thermal expansion, and $V_{0}$ is the volume of the box at the target temperature. The calculated *T*_g_ value for ESO100-85% is 338.5 K. The calculated CTE of ESO100-85% is 2.51 × 10^−4^/K at 300 K (in the rubber state) and 5.57 × 10^−4^/K at 500 K (in the glass state). Additionally, the cooling rates used in MD simulations were considerably higher than those used in experiments. To examine the sensitivity of ESO-based thermosetting resins to cooling rate, four cooling rates (20 K/100 ps, 20 K/300 ps, 20 K/600 ps, and 20 K/1500 ps) were applied to the ESO100-85% system.

### 2.5. Deforming

**Figure 4:**
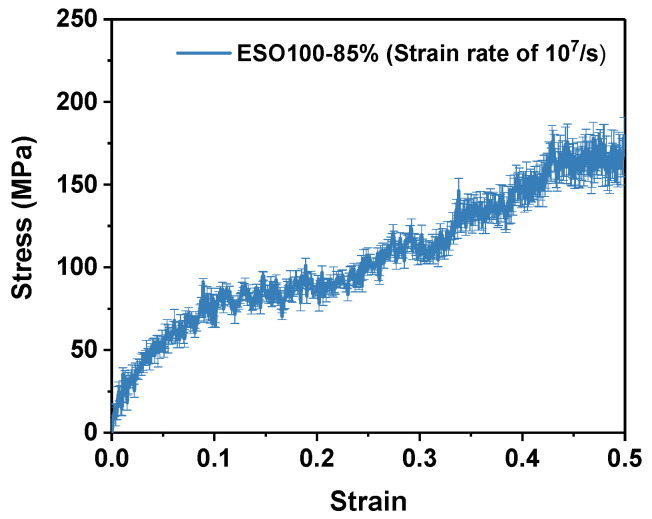
Stress–strain curves for the ESO100-85% system.

The mechanical properties of the DGEBA/ESO blend epoxy resin were investigated through uniaxial deformation at 300 K using the LAMMPS “fix deform” method. The deformation was conducted under the NPT ensemble with a constant strain rate of 10^7^/s, while atmospheric pressure was maintained in the non-tensile direction. Uniaxial deformation was performed on three samples in the x, y, and z directions to eliminate differences in the samples and structural variations in different directions. To investigate the strain rate effect of the ESO-based epoxy resin, three rates (10^7^/s, 10^8^/s, and 10^9^/s) were selected to deform ESO100-85%. Figure 4 shows the average stress–strain curve and the standard error for the ESO100-85% system at a strain rate of 10^7^/s. 

## 3. Results and Discussion

### 3.1. Evolution of Cross-Linking Network

**Figure 5:**
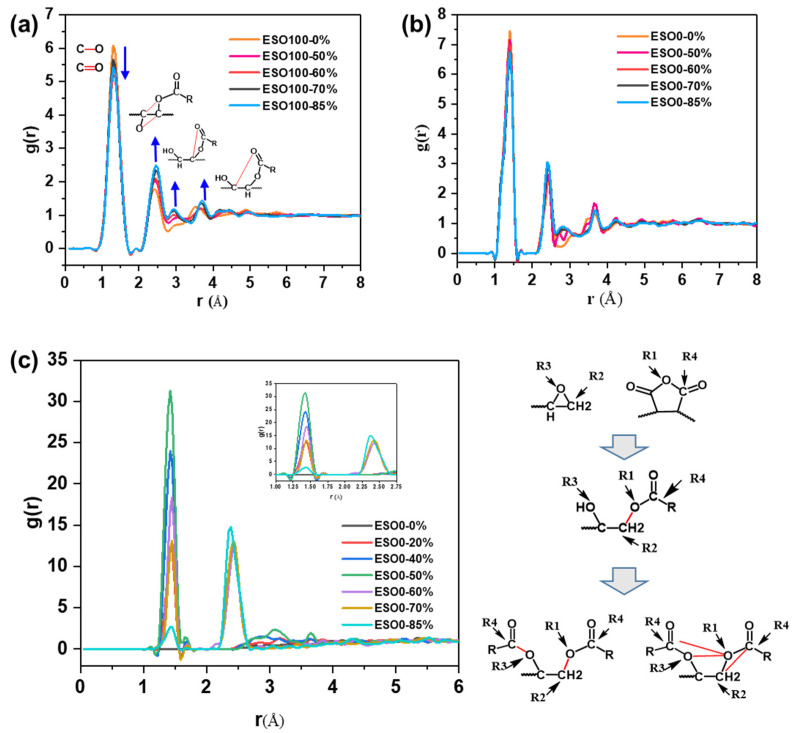
(**a**) RDF of “C” atoms and “O” atoms in ESO100; (**b**) RDF of “C” atoms and “O” atoms in ESO0; and (**c**) RDF of “R2/R3” atoms and “R1/R4” atoms in ESO0.

For the cross-linking system, the evolution of network structure is usually determined by analyzing the radial distribution function (RDF). Figure 5a–c display the RDFs for O atom, C atom, and pairs of reactive atoms in the cross-linking network. Figure 5a,b show that the peak corresponding to the lengths of the C–O and C=O bonds at around 1.3 Å decrease as the cross-linking degree increases. Meanwhile, the peaks at around 2.3 Å (C–C–O sequence length) and 3.0 Å (O=C–O–C sequence length) and 3.6 Å (O=C–O–C–C sequence length) increase with the cross-linking degree. Figure 5c shows the RDF of “R2/R3” and “R1/R4” atoms at different cross-linking degrees. It can be found that two distinct peaks are located at 1.3 Å and 2.4 Å, respectively. The peak of at 1.3 Å increases and then decreases with the cross-linking degree, while the peak at 2.4 Å occurs after the cross-linking degree reaches 50%. In the early stages of cross-linking, the C(R2)–O(R3) and C(R4)–O(R1) bonds break down to form equal amounts of C(R2)–O(R1) and O(R3)–C(R4) bonds, resulting in an increase in the peak at 1.3 Å. As cross-linking continues, each epoxy group reacts twice to form a O=C(R4)–O(R3)–C–C(R2)–O(R1)–C(R4)=O sequence. The probability of finding a C(R4)–O(R3)–C–C(R2) or C(R2)–O(R1)–C(R4) or O(R3)–C–C(R2)–O(R1) sequence increases. As a result, the peak around 2.4 Å increases, while the peak at 1.3 Å decreases. The results of the RDF analysis indicate that the cross-linking network modeling of anhydride-cured epoxy was successful.

**Figure 6:**
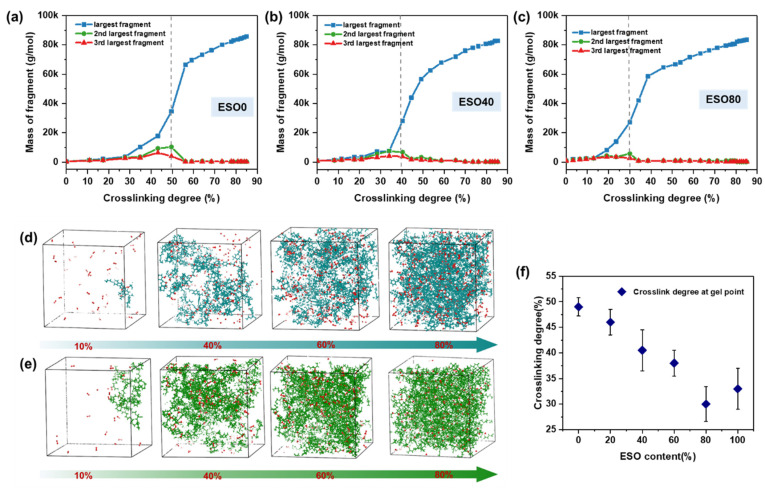
Variation in the mass of the largest, second largest, and third largest molecules with the cross-linking degree in (**a**) ESO0, (**b**)ESO40, and (**c**)ESO80. Evolution of largest molecular cluster with increasing cross-linking degree of (**d**) ESO0 and (**e**) ESO100. (**f**) Gel point corresponding to the ESO content.

Figure 6 illustrates the gelation transition process of the ESO/DGEBA mixed system cured with MHHPA. The gel point can be estimated in the MD simulation by monitoring the molecular weights of the largest and second largest molecule groups during the curing process. Figure 6a, Figure 6b, and Figure 6c display the molecular weights of the largest, second largest, and third largest macromolecules in the ESO0, ESO40, and ESO80 model boxes as a function of cross-linking density, respectively. All the molecular weights of the largest molecule increase significantly, while the molecular weight of the second largest molecule increases to a maximal value and subsequently decreases, where the gel point reaches. Compared to the ESO0 and ESO40 systems, a mutation in the largest molecular weight of ESO80 occurs at lower cross-linking degree. Figure 6d and Figure 6e show the evolution of the largest molecular clusters in the ESO0 and ESO100 systems as the cross-linking degree increases, respectively. As one can find, the size of the largest molecular clusters shows a gradual increase with the cross-linking degree. At a cross-linking degree of 60% in the ESO0 system, a molecule appears that spans the entire simulation box, which is referred to as a gel. In the ESO100 system, a gel is formed at a cross-linking degree of 40%. Thus, as the ESO content in the system increases, there is a tendency for gelation at a lower cross-linking degree, as presented in Figure 6f.

**Figure 7:**
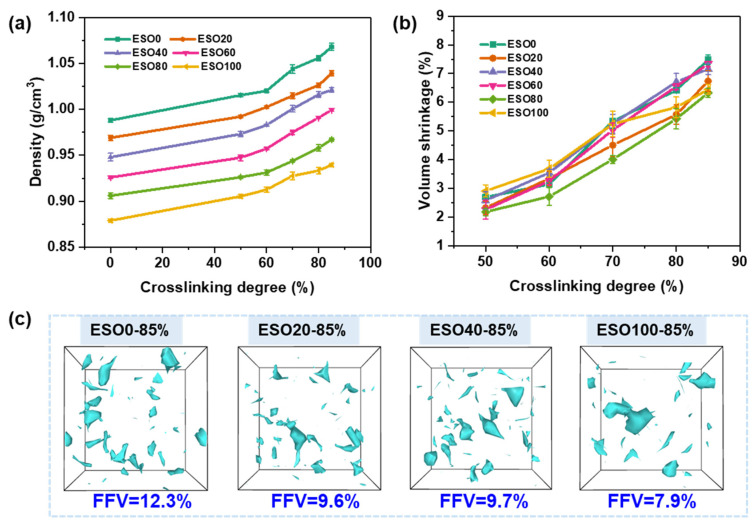
(**a**) Density and (**b**) volume shrinkage corresponding to cross-linking degree at modeling temperature of 500 K. (**c**) Fraction of free volume obtain by OVITO 3.5.1 software (probe radius = 2.6 Å).

Figure 7a,b show the density and volume changes induced by cross-linking at modeling temperature of 500 K. The volumetric shrinkage was calculated as the percent change in the volume of the cross-linked model at a specific cross-linking degree, with respect to the uncross-linked model. It can be clearly seen that the mass density of all systems increases with the increase in the cross-linking degree. The mass density of a system containing a lower ESO content is higher than that of system with a higher ESO content, which can be attributed to the differences in the structures of ESO and DGEBA. The ESO structure contains numerous flexible long carbon chains, whereas the DGEBA structure has a rigid benzene ring structure. As covalent bonds form, both mass density and bulk shrinkage gradually increase with the increase in the cross-linking degree. The highest volumetric shrinkage of 7.48% was observed for the ESO100 system at the cross-linking degree of 85%. Additionally, the mass density of the system rapidly increases after gelation. This is because the system is in the liquid state before gelation, and no obvious shrinkage can be observed during cross-linking. After gelation, the system gradually transforms into a solid state with covalent connections among molecules, resulting in an increase in volume shrinkage.

The fraction of free volume (FFV) of the system was obtained by calculating the ratio of the free volume to the total volume of the cross-linked system. MD simulations have demonstrated that the presence of free volume in the system and the distribution of the size of these cavities are closely related to the macroscopic properties of the thermoset materials [35]. The free volume voids in MHHPA-cured ESO/DGEBA cross-linked networks were captured by OVITO as shown in Figure 7c. At a cross-linking degree of 85%, the FFV of ESO0, ESO20, ESO40, and ESO100 were 12.3%, 9.6%, 9.7%, and 7.9%, respectively. The ESO0 system has the highest free volume fraction but the smallest average hole size. This is because the DGEBA structure contains two benzene rings, which contribute to the high free volume content. The different number and position of epoxy groups in the fatty acid structures of ESO bring about an uneven distribution of cross-linking sites. Thus, the dense cross-linking region generates a large void in the ESO-rich network.

### 3.2. Thermal Properties

#### 3.2.1. Effect of Cooling Rate on Glass Transition Temperature 

**Figure 8:**
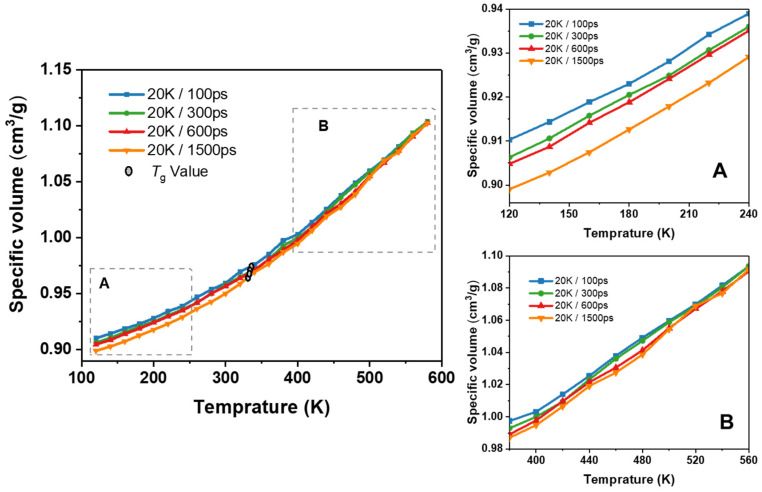
Specific volume–temperature relationships for the ESO100-85% at different cooling rates (region A and region B represent the specific volume–temperature relationships in the glassy state and rubbery state, respectively).

Figure 8 shows the variation in specific volume with temperature for the ESO100-85% system obtained at four cooling rates. The relationship of specific volume and temperature depends on the cooling rate to some extent. During the cooling process, the transition of the system from the rubbery to the glassy state is as follows: (1) at high temperatures, the specific volume has a similar starting point and it is less affected by the cooling rate; (2) as the system temperature gradually decreases, the trend line begins to diverge downward, with slower cooling rates resulting in lower specific volume values at the same temperature (inset B); and (3) when the temperature further decreases, the trend lines of specific volume and temperature exhibit similar slopes even at different cooling rates (inset A), where the system is in the glassy state. It is worthwhile noting that the *T*_g_ values are higher with a faster cooling rate. The *T*_g_ obtained at cooling rates of 20 K/100 ps, 20 K/300 ps, 20 K/600 ps, and 20 K/1500 ps are 334.7 K, 336.2 K, 338.5 K, and 343.2 K, respectively. The simulation results suggest that the cooling rate has little effect on the molecular chain arrangement and mass density of the ESO100-85% system in the rubbery state. Although the particle motion slows down as the temperature decreases, the simulation time is not long enough to reach the corresponding equilibrium chain configuration. This is in agreement with the results obtained by Khare et al. [36].

Table 2 shows the *T*_g_ values obtained from simulations and reported in the literature by experimental method. It should be noted that the *T*_g_ values varies for different measurements, i.e., DSC measurements usually yield lower values than DMA measurements. Differences in the curing process and sample preparation conditions can also affect the properties of epoxy resins, resulting in variations in the experimentally measured *T*_g_ values among researchers. The MD simulation values tend to be slightly higher than experimental values. Although the MD simulation cannot provide a complete and accurate prediction of *T*_g_ for ESO-based thermosetting resins, the obtained trends are considered reliable.

#### 3.2.2. Role of ESO Content and Cross-Linking Degree

**Figure 9:**
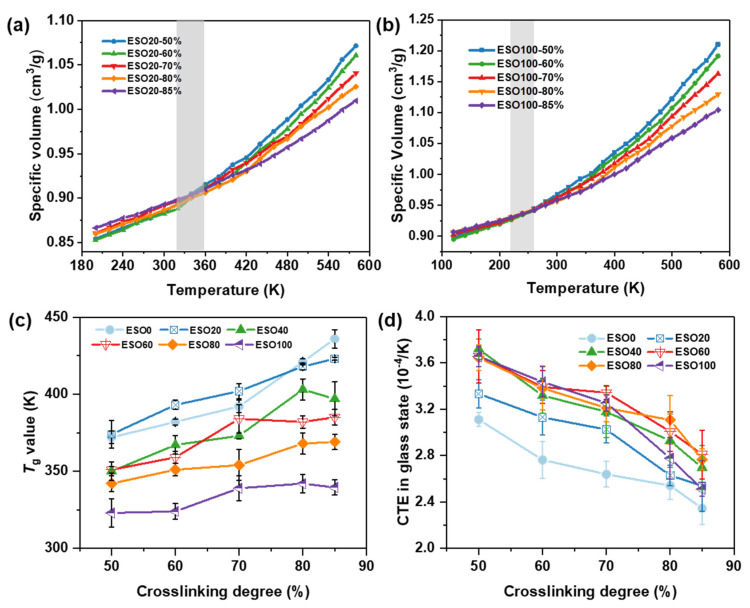
Specific volume–temperature relationships for (**a**) ESO20 and (**b**) ESO100; (**c**) *T_g_* value and (**d**) CTE in the glassy state for each system at different cross-linking degrees.

Figure 9a and Figure 9b illustrate the effect of the cross-linking degree on the specific volume–temperature relationship of ESO20 and ESO100, respectively. At a high temperature, the specific volume of the system with a low cross-linking degree is greater than that of the system with a high cross-linking degree. As the temperature decreases, the specific volume curves of the system with different cross-linking degrees gradually decrease and show a cross point. The system with a low cross-linking degree presents a large temperature-induced volume change. This is related to the topology of the network, where the systems with low cross-linking degrees have more uncross-linked free-moving short chains that can be reoriented even at low temperatures. Conversely, at higher temperatures, the uncross-linked short chains lead to greater volume expansion [40]. Thus, the mass density of ESO20-50% is higher than that of ESO20-85% at lower temperatures, but lower than that of ESO20-85% at higher temperatures.

It is found that the specific volume–temperature curves for ESO20 with different cross-linking degrees intersect between 320 and 360 K, while for ESO100, the intersection occurs between 240 and 280 K. The specific volume of ESO100-85% decreased by 0.18 cm^3^/g from 580 K to 200 K, whereas that of ESO20-85% decreased by 0.14 cm^3^/g. These results suggest that temperature has a more significant effect on the mass density of ESO100. Compared to DGEBA, the epoxy groups of ESO are located in the middle of the chain. The cross-linked structure of the ESO-rich system contains a large number of dangling long carbon chains that cannot participate in the reaction, which provide large volume expansion at a high temperature.

The *T*_g_ value and CTE of each system with different ESO contents and different cross-linking degrees are shown in Figure 9c and Figure 9d, respectively. The *T*_g_ values of each ESO-based thermosetting increase with the increase in cross-linking degree and decrease with the increase in the ESO content. This is because DGEBA is rigid, while ESO monomers contain a flexible long carbon chain. It can be observed that the increase in *T*_g_ due to cross-linking is smaller in the system with a high ESO content, when compared to ESO0 and ESO20. As the ESO content exceeds 40%, the *T*_g_ increases slowly in the interval of cross-linking degree of 70–85%. It is because, once the ESO-based thermosetting reaches a certain degree of cross-linking, the restriction of cross-linking sites on the mobility of molecular chain is no longer significant. However, continuous cross-linking generates more free-volume holes, which increases the activity of unreacted chain segments of ESO. Thus, the *T*_g_ varies with the cross-linking degree, and it is influenced by both cross-linking sites and the free volume fraction. This explains the decrease in *T*_g_ of ESO40 and ESO60 at high cross-linking degrees. Figure 9d shows that the CTE of each system decreases with the increase in the cross-linking degree. ESO0 has a lower CTE value followed by ESO20. The influence of ESO content on the CTE is insignificant when the ESO content exceeds 40%.

### 3.3. Mechanical Properties

#### 3.3.1. Effect of Strain Rate on Mechanical Properties

**Figure 10:**
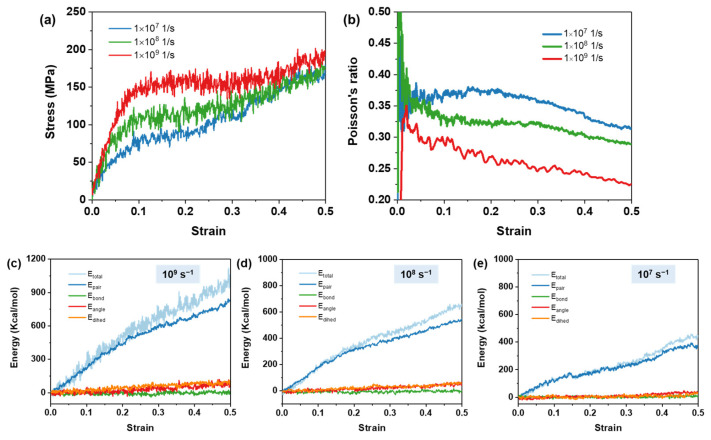
(**a**) Strain–stress curves and (**b**) Poisson’s ratio–strain curves obtained for ESO100-85% at different deformation rates. Energy response of ESO100-85% at deformation rates of (**c**) 10^9^ s^−1^, (**d**) 10^8^ s^−1^, and (**e**) 10^7^ s^−1^.

Figure 10a shows the stress–strain curves of ESO100-85% at three different strain rates under uniaxial deformation. Both yield strength and Young’s modulus show significate increase with the increase in strain rate. The reason is that certain relaxation structures do not have enough time to be activated at a high strain rate, and the network exhibits a delay in response to deformation, leading to the occurrence of excessive stresses. Figure 10b shows the variation in Poisson’s ratio with the strain rate. The Poisson’s ratio with different strain rates fluctuates significantly at the low strains. This is because the atoms rapidly align in the direction of loading, causing severe volume fluctuations. It should be noted that the Poisson’s ratio at the strain rate of 10^7^/s increases with strain before 20% strain, which is due to the onset of plastic flow [40]. After a strain of more than 20%, Poisson’s ratio decreases with the increasing strain, indicating that the properties start to deteriorate. However, Poisson’s ratio at strain rates of 10^8^/s and 10^9^/s decreases during the whole deforming process. Figure 10c–e show the energy response behavior of ESO100-85% at three different strain rates. The energy response curves indicate that the non-bonding energy (*E*_pair_) is the primary contribution to the total energy, while the bonding energy (*E*_bond_) has a negligible effect on the tensile energy. Additionally, the dihedral angular torsional energy (*E*_dhed_) and the angular bending energy (*E*_angle_) only respond to strain at high strain rates. These results demonstrate that a high strain rate restricts network flow. Therefore, strain rates of 10^8^/s and 10^9^/s are unsuitable for the simulation of the mechanical properties of ESO-based thermosets. The deforming rate of 10^7^/s was chosen in the mechanical property simulation. If the deformation rate is further reduced, the computational efficiency would be greatly reduced.

#### 3.3.2. Role of ESO Content and Cross-Linking Degree

**Figure 11:**
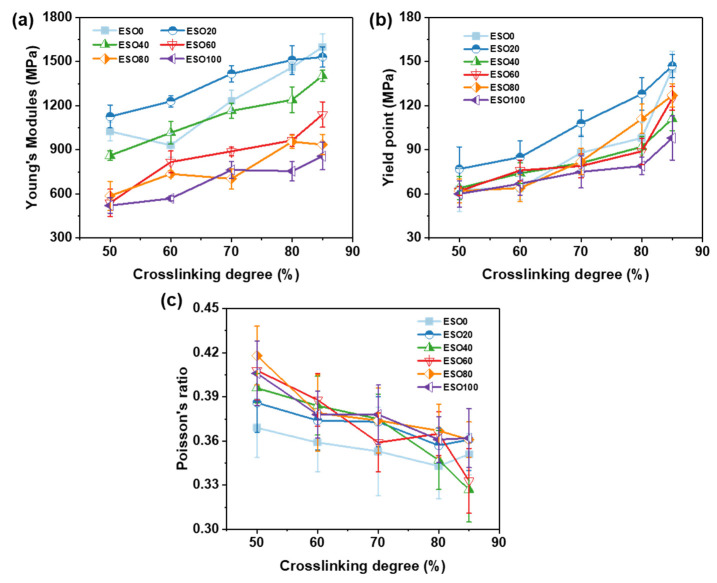
Variation in (**a**) Young’s modulus, (**b**) yield strength, and (**c**) Poisson’s ratio of each system with cross-linking degree.

Uniaxial deformations were also carried out for each system with cross-linking degrees of 50%, 60%, 70%, 80%, and 85% to analyze the effect of ESO content and cross-linking degree on mechanical properties. The Young’s modulus was calculated by the linear regression of the stress–strain curve of 1% to 5% strain. The Poisson’s ratio was determined from the model at 10% strain. The yield strength is defined as the stress at 15% strain where the slope of the stress–strain curve has significantly decreased. The Young’s modulus as a function of the cross-linking degree for systems with different ESO contents is shown in Figure 11a. The Young’s modulus increases as the cross-linking degree increases, as the increase in the network connectivity makes the material harder. The ESO20 system exhibits the highest Young’s modulus at different cross-linking degree except 85%. At 85% cross-linking degree, ESO0 exhibits the highest Young’s modulus of 1645.2 MPa. All systems show a similar increase in the Young’s modulus within the 50–85% range of cross-linking degree. Figure 11b displays the yield strength as a function of the cross-linking degree. At 50% cross-linking degree, the yield strengths of the systems are around 60 MPa. However, at higher cross-linking degrees, the yield strengths of the systems vary significantly from each other. It suggests that the systems exhibit flexibility at lower cross-linking degrees, allowing the molecules to reorient and absorb the deformation energy. Therefore, the structural units that make up the network have little effect on the yield strengths at 50% of cross-linking degree. Conversely, the low flexibility of the highly cross-linked system only allows limited reorientation and slide of the network; thus, the yield strengths show an obvious difference at a high cross-linking degree. Figure 11c shows the Poisson’s ratio as a function of the cross-linking degree for each system. The Poisson’s ratio of each system reduces with the increase in the cross-linking degree. The ESO0 system has a relatively lower Poisson’s ratio than other systems. However, there is no clear pattern found for the effect of the ESO content on Poisson’s ratio when comparing the other systems. Notably, the impact of the cross-linking degree on the Poisson’s ratio appears to surpass that of the ESO content variations.

To validate the modeling reliability, the Young’s modulus and yield strength for the ESO-based thermoset were compared with the values obtained from experimental measurements in studies, as listed in Table 3. One can find that the simulated and experimental values of both Young’s modulus and yield strength are in the same order of magnitude. The simulated Young’s modulus of ESO80 and ESO100 exceeded the experimental values, while those of ESO0 and ESO20 were smaller than experimental values. All the simulated values of yield strength are a bit higher than those from experiments. The huge stain rate difference from actual experiments is the main reason for the prediction error, and the system with a higher ESO content is more affected by the strain rate. Furthermore, the higher simulated values for ESO100 and ESO80 can be attributed to the experimentally low reactivity of the epoxy groups in ESO and the lower cross-linking degree of the thermoset material. In fact, it has always been a challenge to quantitatively predict the values of mechanical properties of thermoset materials, but the trends obtained from simulation predictions are reliable. The results suggest that this modeling method can be expected to be used in other epoxy resin systems. 

## 4. Conclusions

This study utilized the MD simulation method to explore the thermo-mechanical properties of MHHPA-cured DGEBA/ESO blend epoxy resins and variation in the ESO content and the cross-linking degree. The extent of the effect of cooling rate on the glass transition temperature and the deforming rate on the tensile response of the ESO100 system was analyzed by the MD simulation. The simulation results show that the *T*_g_ values are higher for a faster cooling rate, and tensile stresses increase with enlarged strain rates. ESO100 systems have lower free volume fractions compared to systems with a low ESO content but have large cavity size generated by an uneven distribution of cross-linking sites in the ESO-rich region. Moreover, systems with a low cross-linking degree and a low ESO content have large temperature-induced volume changes. The MD simulations also demonstrated that mass density, glass transition temperature, Young’s modulus, and yield strength increase with the increase in the cross-linking degree. However, cross-linking leads to a decrease in Poisson’s ratio and the CTE. When the content of ESO exceeds 40%, the cross-linking degree has significantly higher effects on the CTE and Poisson’s ratio of the system than the chemical components. In general, the predicted properties at different cross-linking degrees offer insight into the evolution of thermoset properties during the curing process. This provides a scientific basis for optimizing bio-based thermosets.

## Figures and Tables

**Table 1:** The composition of simulated systems.

Sample	Mass Ratio of ESO:DGEBA	Number of ESO	Number of DGEBA	Number of MHHPA	Number of MHHPA-OH	Total Number of Atoms
ESO0	0:100	0	165	300	13	13,462
ESO20	20:80	12	135	290	13	14,666
ESO40	40:60	24	99	270	12	13,604
ESO60	60:40	42	68	272	12	14,440
ESO80	80:20	54	35	255	11	14,174
ESO100	100:0	60	0	220	10	13,860

**Table 2:** Thermal properties of each system.

System	MD Predicted *T*_g_ Value/°C	Experimental *T*_g_ Value in the Literature/°C
ESO0-85%	159.5	156 [15], 128 [37], 109.5 [38]
ESO20-85%	149.5	143 [15]
ESO100-85%	61.3–68.1	76 [15], 49–60.8 [10], 68–70 [9], 53–69.8 [39]

**Table 3:** Experimental and simulated values of mechanical properties of each system.

Systems	Properties (MPa)	MD Simulated Values	Experimental Values
ESO0	Young’s modulus	867.3–1645.2	1845.2 ± 50 [15]
	yield strength	63.1–144.4	42.9 ± 2 [15]
ESO20	Young’s modulus	1136.1–1511.4	1779.3 ± 10 [15]
	yield strength	77.0–146.2	48.6 ± 1 [15]
ESO40	Young’s modulus	861.3–1403.0	-
	yield strength	64.1–111.1	-
ESO60	Young’s modulus	744.1–1333.2	1025 ± 18 [41]
	yield strength	62.1–124.5	25.0 [41]
ESO80	Young’s modulus	587.1–955.1	875.7 ± 13 [41]
	yield strength	62.4–128.7	18.5 ± 1 [41]
ESO100	Young’s modulus	490.3–802.9	319.6 ± 17 [41], 351.9 ± 22 [15], 224.7 ± 27 [9]
	yield strength	59.8–97.6	13.7 ± 1 [41], 13 ± 2 [15]

## Data Availability

The raw data supporting the conclusions of this article will be made available by the authors on request.

## Supplementary Materials

The following supporting information can be downloaded at: https://www.mdpi.com/article/10.3390/polym16091229/s1, Figure S1: The composition of ESO in each system; Table S1: The percentage of fatty acids in each system.
