# Supplementary material for: Effects of Chemical Composition and Cross-Linking Degree on the Thermo-Mechanical Properties of Bio-Based Thermosetting Resins: A Molecular Dynamics Simulation Study
Source: Polymers (Basel). 2024 Apr 28;16(9):1229. doi: 10.3390/polym16091229 (PMC11085128; doi:10.3390/polym16091229)
Supplement: Supplementary file 1 [file polymers-16-01229-s001.zip › polymers-2962364-supplementary.pdf]

Effects of Chemical Composition and Cross-Linking Degree on the Thermo-Mechanical Properties of Bio-Based Thermosetting Resins: A Molecular Dynamics Simulation Study

Qiuyu Tang [1], Jie Jiang [1,2,*], Jinjin Li [1], Ling Zhao [1,3] and Zhenhao Xi [1,3,*]

[1] State Key Laboratory of Chemical Engineering, School of Chemical Engineering, East China University of Science and Technology, Shanghai 200237, China; qiuyu_hl@163.com (Q.T.); lijinjin@ecust.edu.cn (J.L.); zhaoling@ecust.edu.cn (L.Z.)

[2] Shanghai Key Laboratory of Advanced Polymeric Materials, School of Materials Science and Engineering, East China University of Science and Technology, Shanghai 200237, China

[3] Shanghai Key Laboratory of Multiphase Materials Chemical Engineering, East China University of Science and Technology, Shanghai 200237, China

* Correspondence: jiangjie@ecust.edu.cn (J.J.); zhhxi@ecust.edu.cn (Z.X.)

The six molecular structures of EVO are named as a1, a2, a3, a4, a5, a6, as shown in Figure 1. The numbers of the six structures in ESO20, ESO40, ESO60, ESO80, ESO100 are shown in Table S1. The percentage of fatty acids in each system is shown in Figure S1.

Table S1. The composition of ESO in each system.

	No. of a1	No. of a2	No. of a3	No. of a4	No. of a5	No. of a6	Total No. of EVO	Epoxy functionalities
ESO0	0	0	0	0	0	0	0	-
ESO20	2	2	3	0	4	1	12	4.07
ESO40	5	3	6	1	9	2	26	4.07
ESO60	8	7	9	1	14	3	42	4.07

ESO80	10	8	12	2	18	4	54	4.07
ESO100	12	8	14	2	19	5	60	4.03

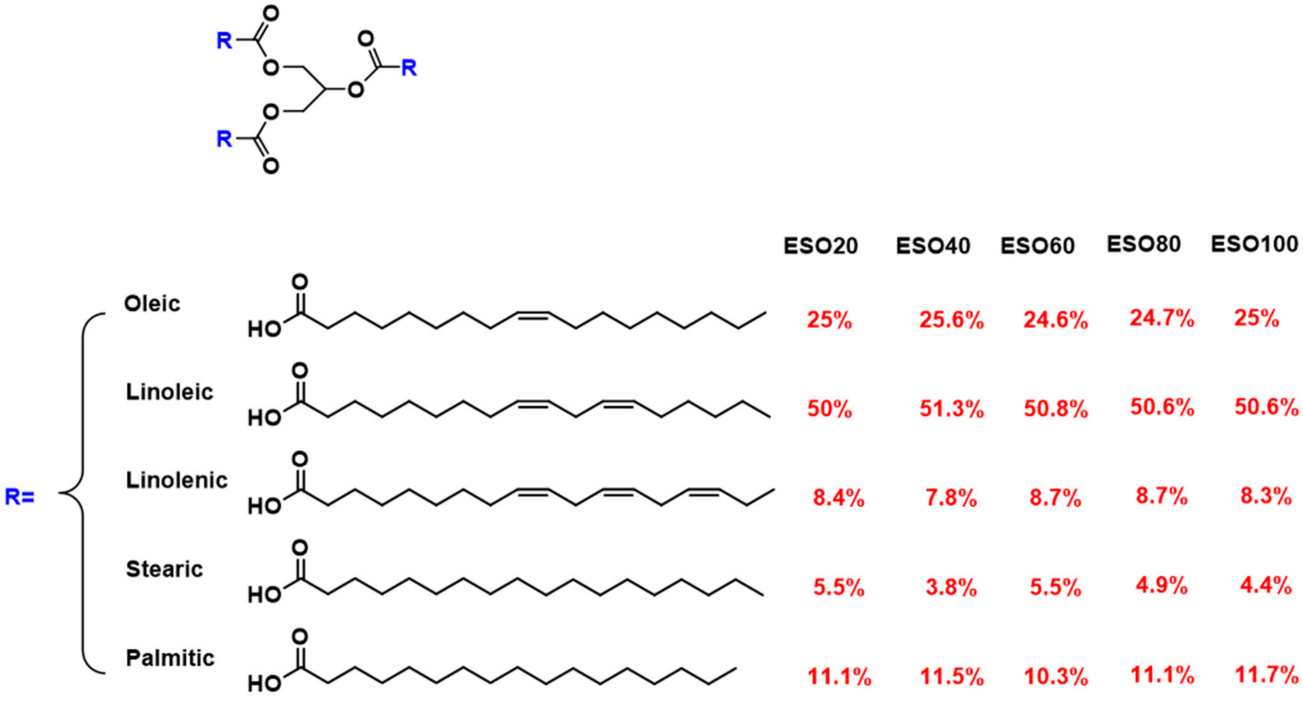

Figure S1. The percentage of fatty acids in each system.
